# Comparison of *R*-ketamine and rapastinel antidepressant effects in the social defeat stress model of depression

**DOI:** 10.1007/s00213-016-4399-2

**Published:** 2016-08-04

**Authors:** Bangkun Yang, Ji-chun Zhang, Mei Han, Wei Yao, Chun Yang, Qian Ren, Min Ma, Qian-Xue Chen, Kenji Hashimoto

**Affiliations:** 1 Division of Clinical Neuroscience, Chiba University Center for Forensic Mental Health, Chiba, 260-8670 Japan; 2 Department of Neurosurgery, Renmin Hospital of Wuhan University, Wuhan, Hubei People’s Republic of China

**Keywords:** Antidepressant, Brain-derived neurotrophic factor, *R*-ketamine, Rapastinel, Synaptogenesis

## Abstract

**Rationale:**

The *N*-methyl-d-aspartate (NMDA) receptor antagonists, including *R*-ketamine and rapastinel (formerly GLYX-13), show rapid antidepressant effects in animal models of depression.

**Objective:**

We compared the rapid and sustained antidepressant effects of *R*-ketamine and rapastinel in the social defeat stress model.

**Results:**

In the tail suspension and forced swimming tests, *R*-ketamine (10 mg/kg, intraperitoneal (i.p.)) or rapastinel (10 mg/kg, i.p.) significantly attenuated the increased immobility time in the susceptible mice, compared with the vehicle-treated group. In the sucrose preference test, both compounds significantly enhanced the reduced preference in susceptible mice 2, 4, or 7 days after a single injection. All mice were sacrificed 8 days after a single injection. Western blot analyses showed that *R*-ketamine, but not rapastinel, significantly attenuated the reduced brain-derived neurotrophic factor (BDNF)-TrkB signaling, postsynaptic density protein 95 (PSD-95), and GluA1 (a subtype of α-amino-3-hydroxy-5-methyl-4-isoxazolepropionic acid (AMPA) receptor) in the prefrontal cortex, dentate gyrus, and CA3 of the hippocampus in the susceptible mice. In contrast, both compounds had no effect against the increased BDNF-TrkB signaling, PSD-95, and GluA1 seen in the nucleus accumbens of susceptible mice. Moreover, sustained antidepressant effect of *R*-ketamine (3 mg/kg, intravenous (i.v.)), but not rapastinel (3 mg/kg, i.v.), was detected 7 days after a single dose.

**Conclusions:**

These results highlight *R*-ketamine as a longer lasting antidepressant compared with rapastinel in social defeat stress model. It is likely that synaptogenesis including BDNF-TrkB signaling in the prefrontal cortex (PFC) and hippocampus may be required for the mechanisms promoting this sustained antidepressant effect.

## Introduction

Depression is one of the most common psychiatric disorders and the leading cause of disability worldwide. Although antidepressants such as selective serotonin reuptake inhibitors (SSRIs) and serotonin norepinephrine reuptake inhibitors (SNRIs) are generally effective in the treatment of this disorder, it can still take weeks before patients feel the full therapeutic benefits. Despite the efficacy of standard treatments, approximately two-thirds of patients with depression fail to respond to pharmacotherapy. Therefore, the development of novel drugs capable of inducing rapid and robust antidepressant responses in treatment-resistant depressed patients is required (Chaki and Fukumoto, 2015; Hashimoto 2015; Monteggia and Zarate 2015).

The *N*-methyl-d-aspartate (NMDA) receptor antagonist ketamine is one of the most attractive antidepressants for treatment-resistant depression (Aan Het Rot et al. 2012; Chaki and Fukumoto, 2015; Krystal et al. 2013; Hashimoto 2009; Hashimoto et al. 2013; Lodge and Mercier, 2015; Sanacora and Schatzberg 2015; Yang and Hashimoto 2014). A single sub-anesthetic dose (0.5 mg/kg) of ketamine produces a rapid and robust antidepressant response in two-thirds of patients with treatment-resistant depression, which can last for over a week (Aan Het Rot et al. 2012; Berman et al. 2000; Diazgranados et al. 2010; Zarate et al. 2006; 2012). Ketamine is a racemic mixture containing equal parts of *R*-ketamine and *S*-ketamine (esketamine). Esketamine has an approximately fourfold greater affinity for the NMDA receptor than the *R*-ketamine (Domino 2010). Singh et al. (2016) reported that an infusion of esketamine caused a rapid antidepressant effect in treatment-resistant depressed patients, although psychotomimetic side effects were the highest at 40 min after a single infusion. In contrast, *R*-ketamine appears to be a potent, long-lasting, and safe antidepressant, relative to esketamine, since *R*-ketamine may appear to be free of side effects such as psychosis and abuse liability (Hashimoto 2014, 2016a, 2016b; Yang and Hashimoto 2014; Yang et al. 2015b, 2016; Zhang et al. 2014). However, a clinical study of *R*-ketamine in patients with depression has not yet been reported.

Rapastinel (formerly GLYX-13), a partial agonist at glycine site of the NMDA receptor, shows antidepressant-like effects without ketamine-like side effects in animal models (Burgdorf et al. 2013; Moskal et al. 2014; 2016). A recent double-blind, placebo-controlled study demonstrated that a single intravenous (i.v.) infusion of rapastinel (5 or 10 mg/kg) produced rapid and sustained antidepressant effects in depressed patients who had not responded to another antidepressant and that this drug did not elicit psychotomimetic or other significant side effects (Preskorn et al. 2015). Rapastinel was granted the Fast Track designation by the US FDA in 2014.

The purpose of this study was to compare the antidepressant effects of *R*-ketamine and rapastinel in the social defeat stress model of depression. In addition, we performed Western blot analysis of brain-derived neurotrophic factor (BDNF)-TrkB pathway, postsynaptic density protein 95 (PSD-95), and the α-amino-3-hydroxy-5-methyl-4-isoxazolepropionic acid (AMPA) receptor (GluA1), both of which are required for synaptic plasticity in selected brain regions, since BDNF-TrkB signaling and synaptogenesis are implicated in the mechanisms of rapid antidepressant action of the NMDA receptor antagonists (Autry et al. 2011; Duman and Aghajanian 2012; Lepack et al. 2014; Ohgi et al. 2015; Yang et al. 2015b).

## Materials and methods

### Animals

Male adult C57BL/6 mice, aged 8 weeks (body weight 20–25 g, Japan SLC, Inc., Hamamatsu, Japan), and male adult CD1 mice, aged 13–15 weeks (body weight >40 g, Japan SLC, Inc., Hamamatsu, Japan), were used in experiments. Animals were housed under controlled temperatures and 12 h light/dark cycles (lights on between 07:00 and 19:00 h), with ad libitum food and water. This study was carried out in strict accordance with the recommendations in the Guide for the Care and Use of Laboratory Animals of the National Institutes of Health. The protocol was approved by the Chiba University Institutional Animal Care and Use Committee. All efforts were made to minimize suffering.

### Drugs and drug administration

**Fig. 1:**
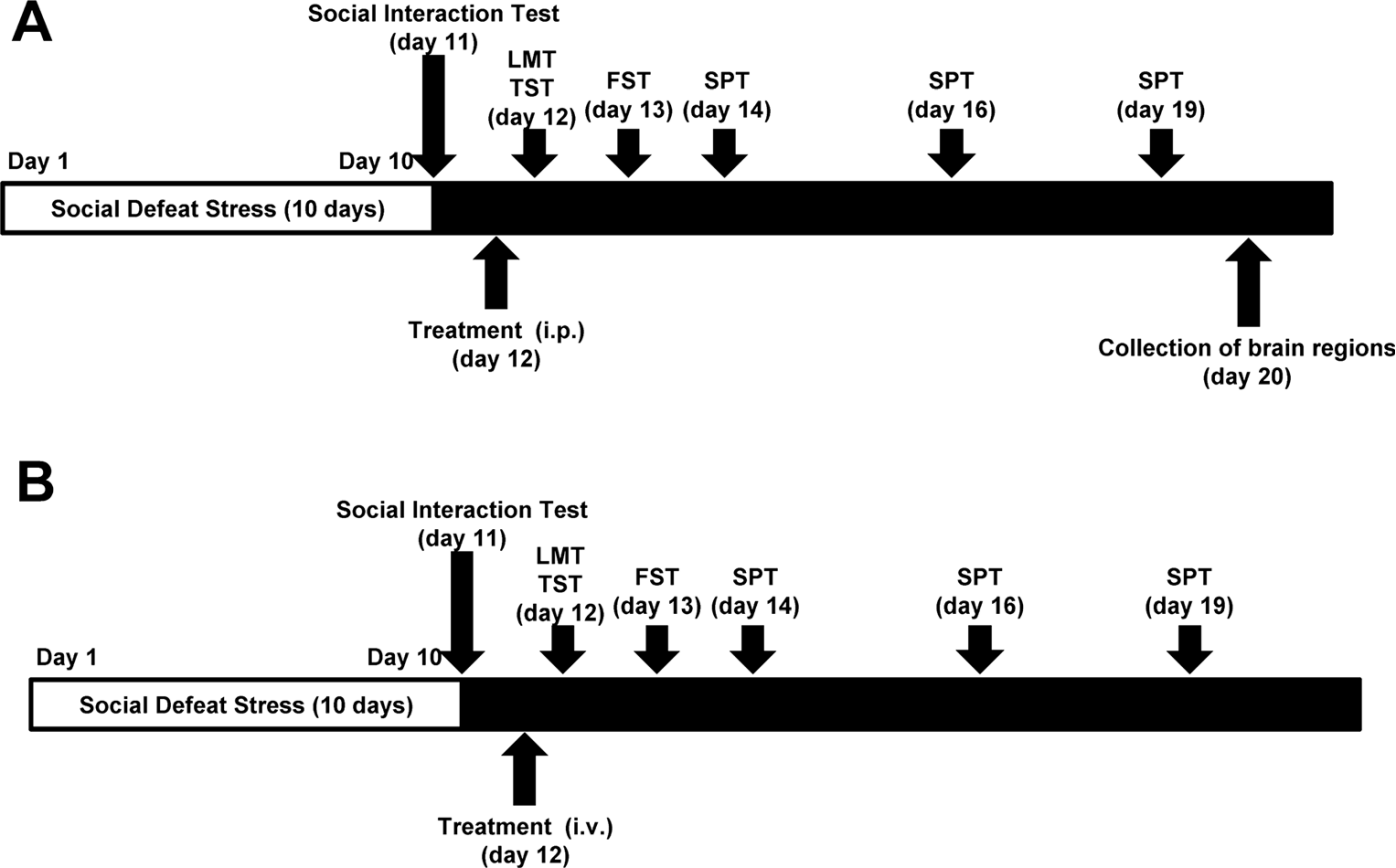
The schedule of social defeat stress, drug administration, behavioral tests, and brain sampling. **a** Repeated social defeat stress was performed for 10 days (day 1–day 10). Social interaction test was performed on day 11, and susceptible mice were used in the subsequent experiments. Vehicle (10 ml/kg, i.p.), *R*-ketamine (10 mg/kg, i.p.), or rapastinel (10 mg/kg, i.p.) was administered (day 12). Locomotion test (*LMT*) and tail suspension test (*TST*) were performed 2 and 4 h after a single injection, respectively (day 12). Forced swimming test (*FST*) was performed 1 day after injection (day 13). One percent sucrose preference test (*SPT*) was performed 2 days (day 14), 4 days (day 15), and 7 days (day 19) after injection. Collection of brain regions was performed at day 20. **b** Repeated social defeat stress was performed for 10 days (day 1–day 10). Social interaction test was performed on day 11, and susceptible mice were used in the subsequent experiments. Vehicle (5 ml/kg, i.v.), *R*-ketamine (3 mg/kg, i.v.), or rapastinel (3 mg/kg, i.v.) was administered (day 12). LMT and TST were performed 2 and 4 h after a single injection, respectively, (day 12). FST was performed 1 day after injection (day 13). One percent SPT was performed 2 days (day 14), 4 days (day 15), and 7 days (day 19) after a single injection

*R*-ketamine hydrochloride was prepared by recrystallization of racemic ketamine (Ketalar®, ketamine hydrochloride, Daiichi Sankyo Pharmaceutical Ltd., Tokyo, Japan) and d-(-)-tartaric acid (or l-(+)-tartaric acid), as described previously (Zhang et al. 2014). The purity of these stereoisomers was determined by a high-performance liquid chromatography (CHIRALPAK® IA; column size, 250 × 4.6 mm; mobile phase, *n*-hexane/dichloromethane/diethylamine (75/25/0.1), Daicel Corporation, Tokyo, Japan). On the day of intraperitoneal (i.p.) injection, vehicle (10 ml/kg; distilled water), *R*-ketamine (10 mg/kg as hydrochloride salt), and rapastinel (GLYX-13; 10 mg/kg, Tocris Bioscience, Bristol, UK) were administered into mice (Fig. 1a). The doses of *R*-ketamine (10 mg/kg) and rapastinel (10 mg/kg) were selected as reported previously (Lu et al. 2014; Yang et al. 2015b, 2016; Zhang et al. 2014). For i.v. administration experiment, vehicle (5 ml/kg; distilled water), *R*-ketamine (3 mg/kg), and rapastinel (3 mg/kg) were administered into mice (Fig. 1b). The dose of rapastinel (3 mg/kg) was selected as reported previously (Burgdorf et al. 2013).

### Social defeat procedure

The social defeat procedure was performed as previously reported (Berton et al. 2006; Golden et al. 2011; Ren et al. 2016; Yang et al. 2015b; Zhang et al. 2015b). Every day, the C57BL/6 mice were exposed to a different CD1 aggressor mouse for 10 min, total for 10 days. When the social defeat session ended, the resident CD1 mouse and the intruder mouse were housed in one half of the cage separated by a perforated Plexiglas divider to allow visual, olfactory, and auditory contact for the remainder of the 24-h period. At 24 h after the last session, all mice were housed individually. On day 11, a social interaction test was performed to identify subgroups of mice that were susceptible and unsusceptible to social defeat stress (Fig. 1). This was accomplished by placing mice in an interaction test box (42 × 42 cm) with an empty wire mesh cage (10 × 4.5 cm) located at one end. The movement of the mice was tracked for 2.5 min, followed by 2.5 min in the presence of an unfamiliar aggressor confined in the wire mesh cage. The duration of the subject’s presence in the “interaction zone” (defined as the 8-cm-wide area surrounding the wire mesh cage) was recorded by a stopwatch. The interaction ratio was calculated as time spent in an interaction zone with an aggressor/time spent in an interaction zone without an aggressor. An interaction ratio of 1 was set as the cutoff: mice with scores <1 were defined as “susceptible” to social defeat stress and those with scores ≥1 were defined as “unsusceptible.” Only susceptible mice were used in the subsequent experiments.

### Behavioral tests

Behavioral tests were performed as reported previously (Ren et al. 2016; Yang et al. 2015b; Zhang et al. 2015b).

#### Locomotion

The locomotor activity was measured by an animal movement analysis system SCANETMV-40 (MELQUEST Co., Ltd., Toyama, Japan), and the mice were placed in experimental cages (length × width × height, 560 × 560 × 330 mm). The cumulative exercise was recorded for 60 min. Cages were cleaned between testing session.

#### Tail suspension test (TST)

A small piece of adhesive tape placed approximately 2 cm from the tip of the tail for mouse. A single hole was punched in the tape, and mice were hung individually, on a hook. The immobility time was recorded for 10 min. Mice were considered immobile only when they hung passively and completely motionless.

#### Forced swimming test (FST)

The FST was tested by an automated forced swim apparatus SCANET MV-40 (MELQUEST Co., Ltd., Toyama, Japan). The mice were placed individually in a cylinder (diameter, 23 cm; height, 31 cm) containing 15 cm of water, maintained at 23 ± 1°C. Immobility time from activity time as (total)–(active) time was calculated by the apparatus analysis software. The immobility time for mouse was recorded for 6 min.

#### Sucrose preference test (SPT)

Mice were exposed to water and 1 % sucrose solution for 48 h, followed by 4 h of water and food deprivation and a 1-h exposure to two identical bottles, one is water and another is 1 % sucrose solution. The bottles containing water and sucrose were weighed before and at the end of this period, and the sucrose preference was determined.

### Western blot analysis of proBDNF, BDNF, TrkB, p-TrkB, PSD-95, and GluA1

Western blot analysis was performed as reported previously (Yang et al. 2015b; Zhang et al. 2015b). Mice were sacrificed and brains were rapidly removed from the skull. Approximately 1-mm-thick coronal sections were cut, and bilateral tissue punches of prefrontal cortex (PFC), nucleus accumbens (NAc), CA1, CA3, and dentate gyrus (DG) of the hippocampus were dissected on ice using a SZ-LED Kenis light microscope (Osaka, Japan) and stored at −80 °C. Basically, tissue samples were homogenized in Laemmli lysis buffer. Aliquots (10 μg) of protein were measured using the DC protein assay kit (Bio-Rad, Hercules, CA) and incubated for 5 min at 95 °C, with an equal volume of 125 mM Tris/HCl, pH 6.8, 20 % glycerol, 0.1 % bromophenol blue, 10 % β-mercaptoethanol, and 4 % sodium dodecyl sulfate, and subjected to sodium dodecyl sulfate polyacrylamide gel electrophoresis, using 10 % mini-gels (Mini-PROTEAN® TGX™ Precast Gel; Bio-Rad, CA, USA). Proteins were transferred onto polyvinylidene difluoride (PVDF) membranes using a Trans Blot Mini Cell (Bio-Rad). For immunodetection, the blots were blocked with 2 % BSA in TBST (TBS + 0.1 % Tween-20) for 1 h at room temperature (RT) and kept with primary antibodies overnight at 4 °C. The following primary antibodies were used: BDNF (1: 200, Cat. no. H-117, Santa Cruz Biotechnology, Inc., CA, USA), phosphor-TrkB (Tyr-706) (1:200; Cat. no. sc135645, Santa Cruz Biotechnology), TrkB (80E3) (1:1000; Cat. no. 4603, Cell Signaling Technology), postsynaptic density protein 95 (PSD-95) (1 μg/ml, Invitrogen, Carlsbad, CA, USA), and AMPA glutamate receptor 1 (GluA1) (1 μg/ml, Abcam, Cambridge, MA, USA). On the next day, blots were washed three times in TBST and incubated with horseradish peroxidase-conjugated anti-rabbit antibody (1:5000) 1 h, at RT. After final three washes with TBST, bands were detected using enhanced chemiluminescence (ECL) plus the Western Blotting Detection system (GE Healthcare Bioscience). The blots then were washed three times in TBST and incubated with the primary antibody directed against β-actin. Images were captured with a Fuji LAS3000-mini imaging system (Fujifilm, Tokyo, Japan), and immunoreactive bands were quantified.

### Statistical analysis

The data are shown as mean ± standard error of the mean (SEM). Analysis was performed using PASW Statistics 20 (formerly SPSS Statistics; SPSS). Comparisons between groups were performed using the one-way analysis of variance (ANOVA), followed by post hoc Fisher’s least significant difference (LSD) test. *P* values less than 0.05 were considered statistically significant.

## Results

### Effects of i.p. administration of *R*-ketamine and rapastinel in the social defeat stress model

Ketamine produces rapid and long-lasting antidepressant effects in the chronic mild stress model and social defeat stress model (Li et al. 2010; Ma et al. 2013; Yang et al. 2015b; Zhang et al. 2015b). In this study, we compared the effects of i.p. administration of *R*-ketamine and rapastinel in the social defeat stress model (Fig. 1a).

**Fig. 2:**
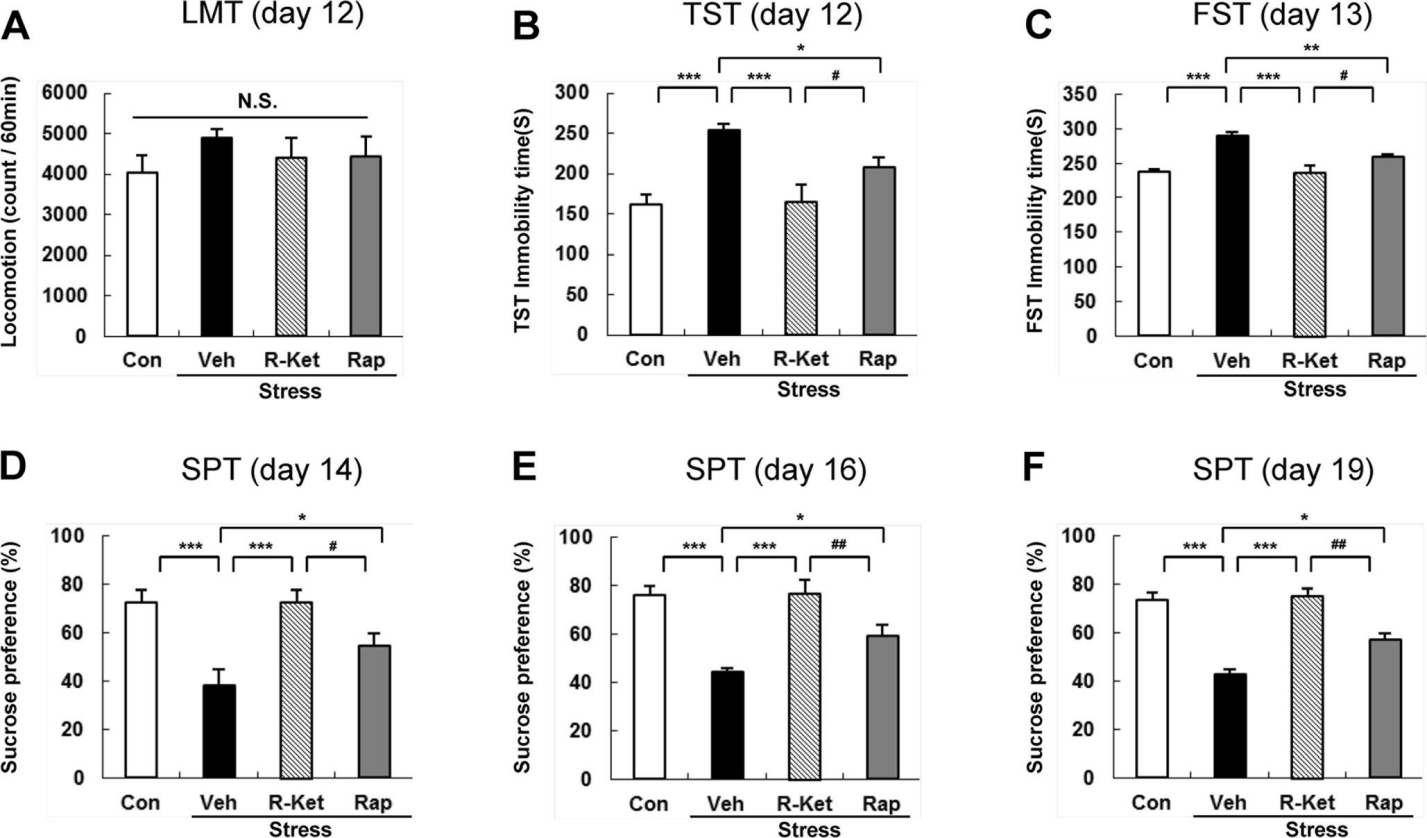
Effects of i.p. administration of *R*-ketamine and rapastinel in social defeat stress model. **a** LMT (day 12), **b** TST (day 12), **c** FST (day 13), **d** SPT (day 14), **e** SPT (day 16), and **f** SPT (day 19). The values represent the mean ± SEM (*n* = 9 or 10). *P < 0.05, **P < 0.01, ***P < 0.001 compared with the vehicle-treated stress group. ^#^ *P* < 0.05, ^##^ *P* < 0.01 compared with *R*-ketamine treated stress group. *N.S.* not significant, *Con* control, *Veh* vehicle, *R-Ket R*-ketamine, *Rap* rapastinel, *LMT* locomotion test, *TST* tail suspension test, *FST* forced swimming test, *SPT* 1 % sucrose preference test

Locomotion showed no difference (*F*_3, 32_ = 0.727, *P* = 0.543) among the four groups (Fig. 2a). In the TST and FST, *R*-ketamine (10 mg/kg, i.p.) and rapastinel (10 mg/kg, i.p.) significantly attenuated the increased immobility times in susceptible mice (Fig. 2b, c). One-way ANOVA detected statistical significance in both the TST and FST (TST: *F*_3, 32_ = 9.839, *P* < 0.001; FST: *F*_3, 32_ = 15.547, *P* < 0.001) among the four groups (Fig. 2b, c). In the SPT, preference of mice after an injection of *R*-ketamine or rapastinel was significantly higher (2D (day 14): *F*_3, 32_ = 8.851, *P* < 0.001, 2E (day 16): *F*_3, 32_ = 15.236, *P* < 0.001, 2F (day 19): *F*_3, 32_ = 14.695, *P* < 0.001) than that of the vehicle-treated group (Fig. 2d–f). In the TST, FST, and SPT, efficacy of *R*-ketamine was more potent than that of rapastinel. These behavioral data suggest that i.p. administration of *R*-ketamine and rapastinel promote rapid antidepressant effects in the social defeat stress model and that *R*-ketamine produces longer lasting antidepressant effects compared to rapastinel.

### Levels of proBDNF and BDNF proteins in the brain regions after i.p. administration of *R*-ketamine or rapastinel

**Fig. 3:**
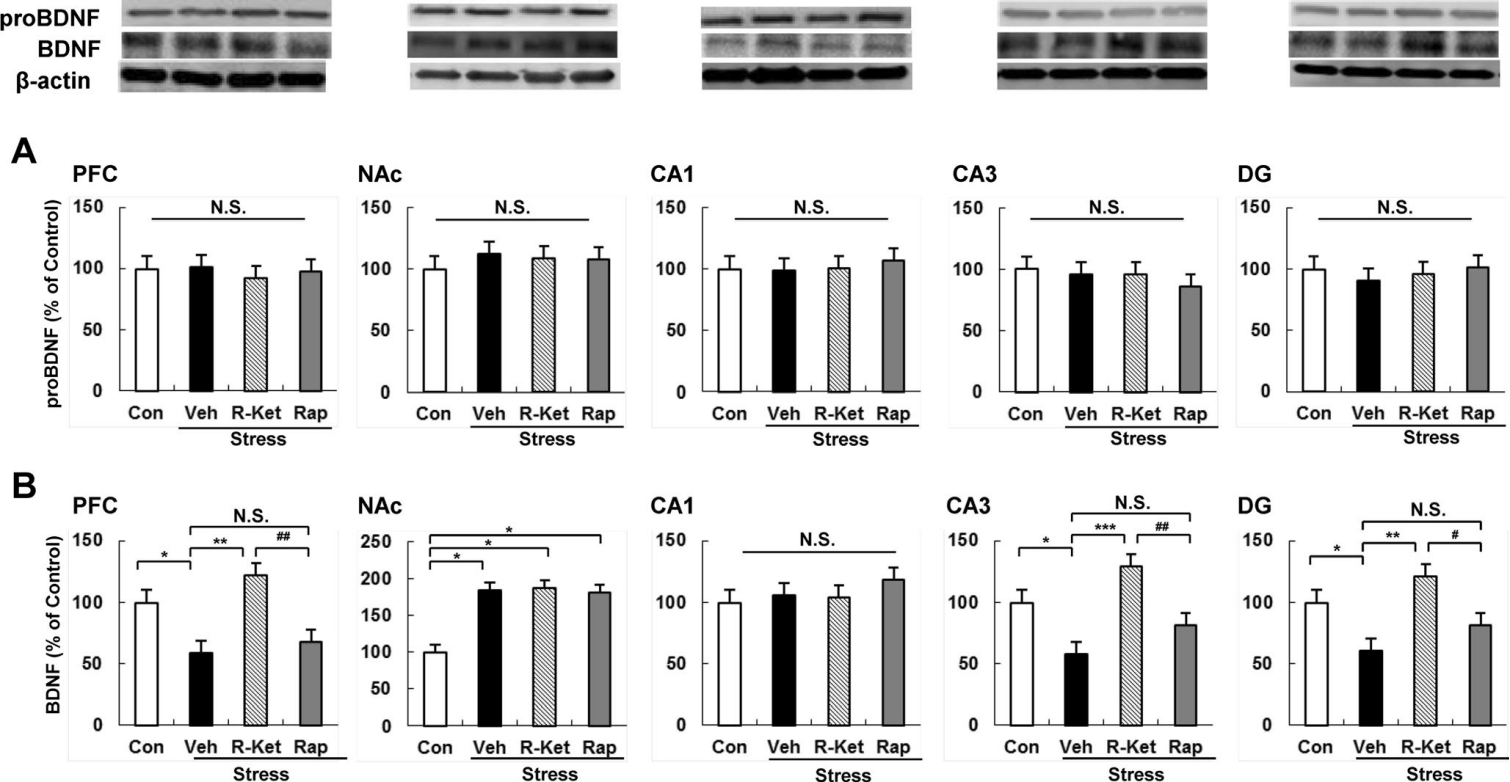
Levels of proBDNF and BDNF in the brain regions. **a** Western blot analysis of proBDNF in PFC, NAc, CA1, CA3, and DG of the hippocampus. The value was expressed as a percentage of that of control mice. The values represent the mean ± SEM (*n* = 6 or 7). *N.S.* not significant. **b** Western blot analysis of BDNF (mature form) in PFC, NAc, CA1, CA3, and DG of the hippocampus. The value was expressed as a percentage of that of control mice. The values represent the mean ± SEM (*n* = 6 or 7). *P < 0.05, **P < 0.01, ***P < 0.001 compared with the vehicle-treated stress group. ^#^ *P* < 0.05, ^##^ *P* < 0.01 compared with *R*-ketamine treated stress group. *N.S.* not significant. *Con* control, *Veh* vehicle, *R-Ket R*-ketamine, *Rap* rapastinel

Since BDNF plays a role in the antidepressant action of ketamine (or *R*-ketamine) (Autry et al. 2011; Lepack et al. 2014; Yang et al. 2015b), we performed Western blot analysis of BDNF and its precursor, proBDNF, in selected brain regions (PFC, NAc, DG, CA1, and CA3 of the hippocampus) 8 days after a single dose of compound (Figs. 1a and 3). One-way ANOVA of proBDNF data showed no significant changes in any test group, for any brain region (Fig. 3a). One-way ANOVA of BDNF data detected statistical significances in all regions, except CA1 [PFC: *F*_3, 20_ = 5.524, *P* = 0.006; NAc: *F*_3, 20_ = 3.118, *P* = 0.049; CA1: *F*_3, 20_ = 0.151, *P* = 0.928; CA3: *F*_3, 20_ = 7.919, *P* = 0.001; DG: *F*_3, 20_ = 6.144, *P* = 0.004] (Fig. 3b). We found that social defeat stress significantly decreased levels of BDNF protein in the PFC, DG, and CA3, but not in CA1, while significantly increasing BDNF protein in the NAc (Fig. 3b), consistent with previous data from the social defeat stress model of depression (Ren et al. 2016; Yang et al. 2015b; Zhang et al. 2015b) and the rat learned helplessness model (Shirayama et al. 2015; Yang et al. 2015a). Interestingly, *R*-ketamine (10 mg/kg) significantly attenuated reduced levels of BDNF protein in the PFC, CA3, and DG 8 days after a single dose, although *R*-ketamine did not affect the increased levels of BDNF protein in the NAc (Fig. 3b). In contrast, rapastinel (10 mg/kg) did not affect alterations in the BDNF levels in any region of susceptible mice (Fig. 3b).

### Levels of TrkB and p-TrkB proteins in the brain regions after i.p. administration of *R*-ketamine or rapastinel

**Fig. 4:**
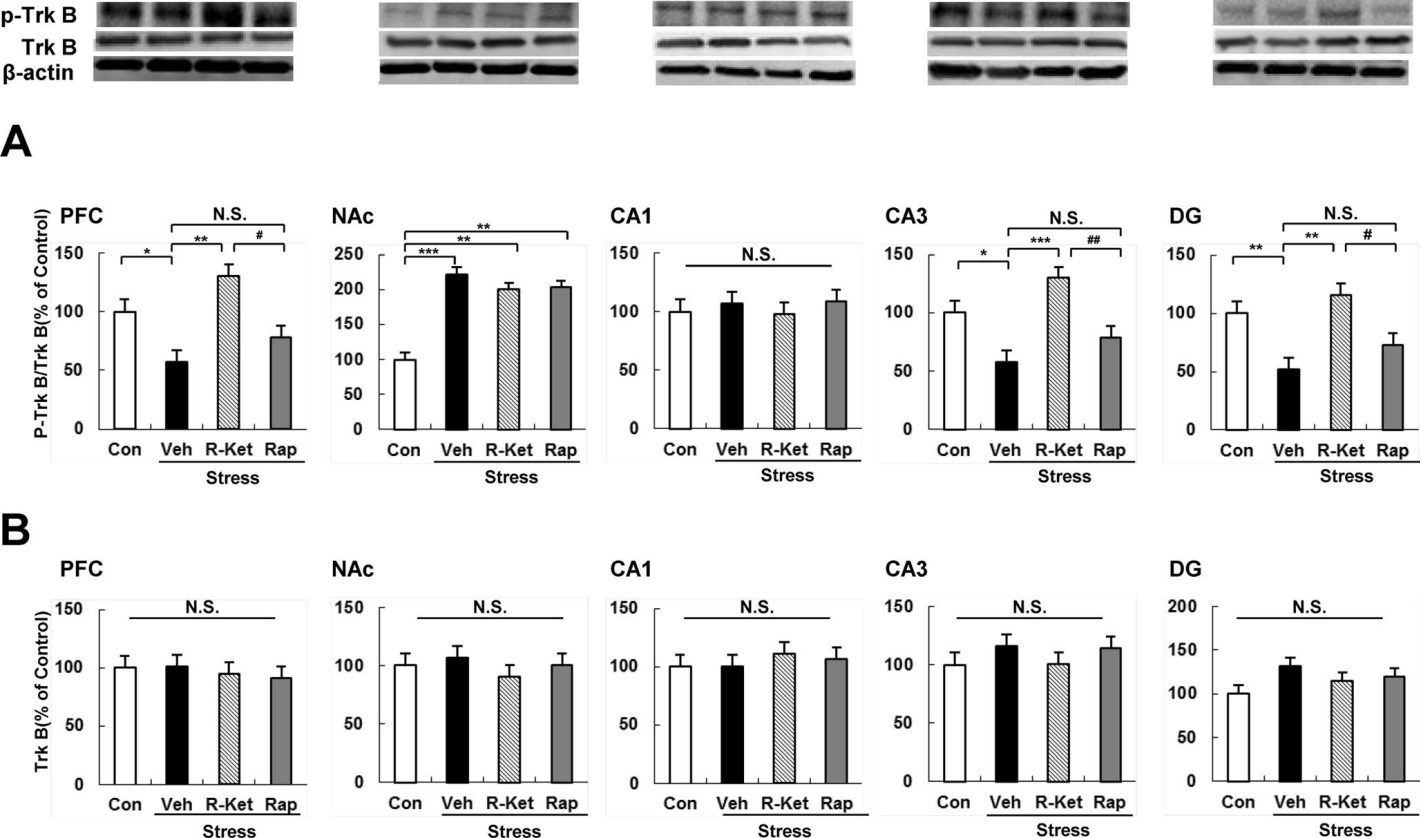
The ratio of p-TrkB/TrkB in the brain regions. **a** Western blot analysis of TrkB and p-TrkB proteins in PFC, NAc, CA1, CA3, and DG of the hippocampus. The value of p-TrkB/TrkB ratio was expressed as a percentage of that of control mice. The values represent the mean± SEM (*n* = 6 or 7). *P < 0.05, **P < 0.01, ***P < 0.001 compared with the vehicle-treated stress group. ^#^ *P* < 0.05, ^##^ *P* < 0.01 compared with *R*-ketamine treated stress group. *N.S.* not significant. **b** The value of total TrkB protein was expressed as a percentage of that of control mice. Values represent the mean ± SEM (*n* = 6 or 7). *N.S.* not significant, *Con* control, *Veh* vehicle, *R-Ket R*-ketamine, *Rap* rapastinel

To clarify whether TrkB activation or inhibition underpins mechanistic action of *R*-ketamine and rapastinel, we performed Western blot analyses of TrkB and phosphorylated TrkB (p-TrkB), an activated form of TrkB, in samples from PFC, NAc, and CA1, CA3, and DG of hippocampus (Fig. 4). One-way ANOVA of p-TrkB/TrkB ratio data showed statistical significances in all regions, except CA1 [PFC: *F*_3, 20_ = 5.279, *P* = 0.008; NAc: *F*_3, 20_ = 7.665, *P* = 0.001; CA1: *F*_3, 20_ = 0.073, *P* = 0.974; CA3: *F*_3, 20_ = 7.791, *P* = 0.001; DG: *F*_3, 20_ = 5.424, *P* = 0.007] (Fig. 4a). Social defeat stress significantly decreased levels of p-TrkB/TrkB ratio in the PFC, DG, and CA3, but not in CA1, while significantly increasing p-TrkB/TrkB ratio in the NAc (Fig. 4a), consistent with previous data from the social defeat stress model of depression (Ren et al. 2016; Yang et al. 2015b; Zhang et al. 2015b). Interestingly, *R*-ketamine (10 mg/kg) significantly attenuated reduced levels of p-TrkB/TrkB ratio in the PFC, CA3, and DG 8 days after a single dose, although *R*-ketamine did not affect the increased levels of the ratio in the NAc (Fig. 4a). In contrast, rapastinel (10 mg/kg) did not affect alterations in the ratio in any region of susceptible mice (Fig. 4a). One-way ANOVA of TrkB protein data showed no significant changes in any test group, for any brain region (Fig. 4b).

### Levels of PSD-95 and GluA1 in selected mouse brain regions after i.p. administration of *R*-ketamine or rapastinel

**Fig. 5:**
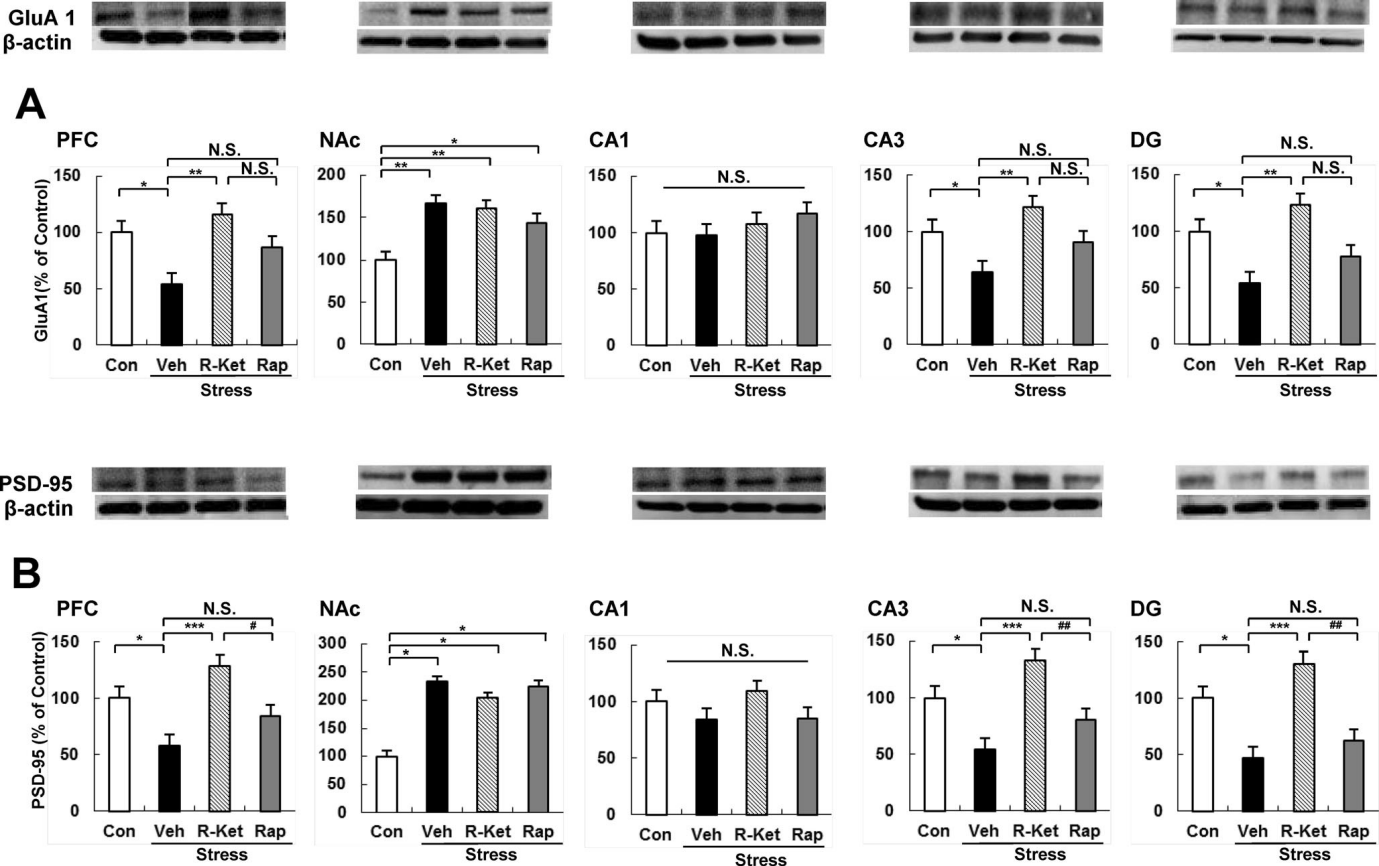
Levels of GluA1 and PSD-95 in the brain regions. **a** Western blot analysis of GluA1 in PFC, NAc, CA1, CA3, and DG of the hippocampus. The value was expressed as a percentage of that of control mice. Values represent the mean ± SEM (*n* = 6 or 7). *P < 0.05, **P < 0.01 compared with the vehicle-treated stress group. *N.S.* not significant. **b** Western blot analysis of PSD-95 in PFC, NAc, CA1, CA3, and DG of the hippocampus. The value was expressed as a percentage of that of control mice. Values represent the mean ± SEM (*n* = 6 or 7). *P < 0.05, **P < 0.01, ***P < 0.001 compared with the vehicle + stress group. ^#^ *P* < 0.05, ^##^ *P* < 0.01 compared with *R*-ketamine treated stress group. *N.S.* not significant, *Con* control, *Veh* vehicle, *R-Ket R*-ketamine, *Rap* rapastinel

We performed Western blot analysis on the synaptogenesis markers, PSD-95 and GluA1, in selected brain regions (Fig. 5). One-way ANOVA of GluA1 data showed statistical significances in all regions, except CA1 [PFC: *F*_3, 20_ = 3.941, *P* = 0.023; NAc: *F*_3, 20_ = 6.713, *P* = 0.003; CA1: *F*_3, 20_ = 0.268, *P* = 0.848; CA3: *F*_3, 20_ = 5.071, *P* = 0.009; DG: *F*_3, 20_ = 3.921, *P* = 0.024] (Fig. 5a). Eight days after a single dose of test drug, social defeat stress significantly decreased levels of GluA1 in the PFC, DG, and CA3 of the hippocampus, but not CA1, whereas it significantly increased the levels of GluA1 in the NAc (Fig. 5a). Interestingly, *R*-ketamine significantly attenuated the reduction of GluA1 protein in the PFC, DG, and CA3 after social defeat stress, although it had no effect on the increased GluA1 levels in the NAc (Fig. 5a). Furthermore, rapastinel did not affect alterations in GluA1 levels following social defeat stress (Fig. 5a).

Next, we performed Western blot analysis of PSD95 in selected mouse brain regions. One-way ANOVA of PSD-95 data showed statistical significances in all regions, except CA1 [PFC: *F*_3, 20_ = 6.992, *P* = 0.002; NAc: *F*_3, 20_ = 3.337, *P* = 0.040; CA1: *F*_3, 20_ = 1.004, *P* = 0.411; CA3: *F*_3, 20_ = 7.922, *P* = 0.001; DG: *F*_3, 20_ = 8.229, *P* = 0.001] (Fig. 5b). Eight days after a single dose of test drug, social defeat stress significantly decreased levels of PSD-95 in the PFC, DG, and CA3 of the hippocampus, but not CA1, whereas it significantly increased the levels of PSD-95 in the NAc (Fig. 5b). Interestingly, *R*-ketamine significantly attenuated the reduction of PSD-95 protein in the PFC, DG, and CA3 after social defeat stress, although it had no effect on the increased PSD-95 levels in the NAc (Fig. 5b). Furthermore, rapastinel did not affect alterations in GluA1 levels in the brain regions following social defeat stress (Fig. 5b).

### Effects of i.v. administration of *R*-ketamine and rapastinel in the social defeat stress model

**Fig. 6:**
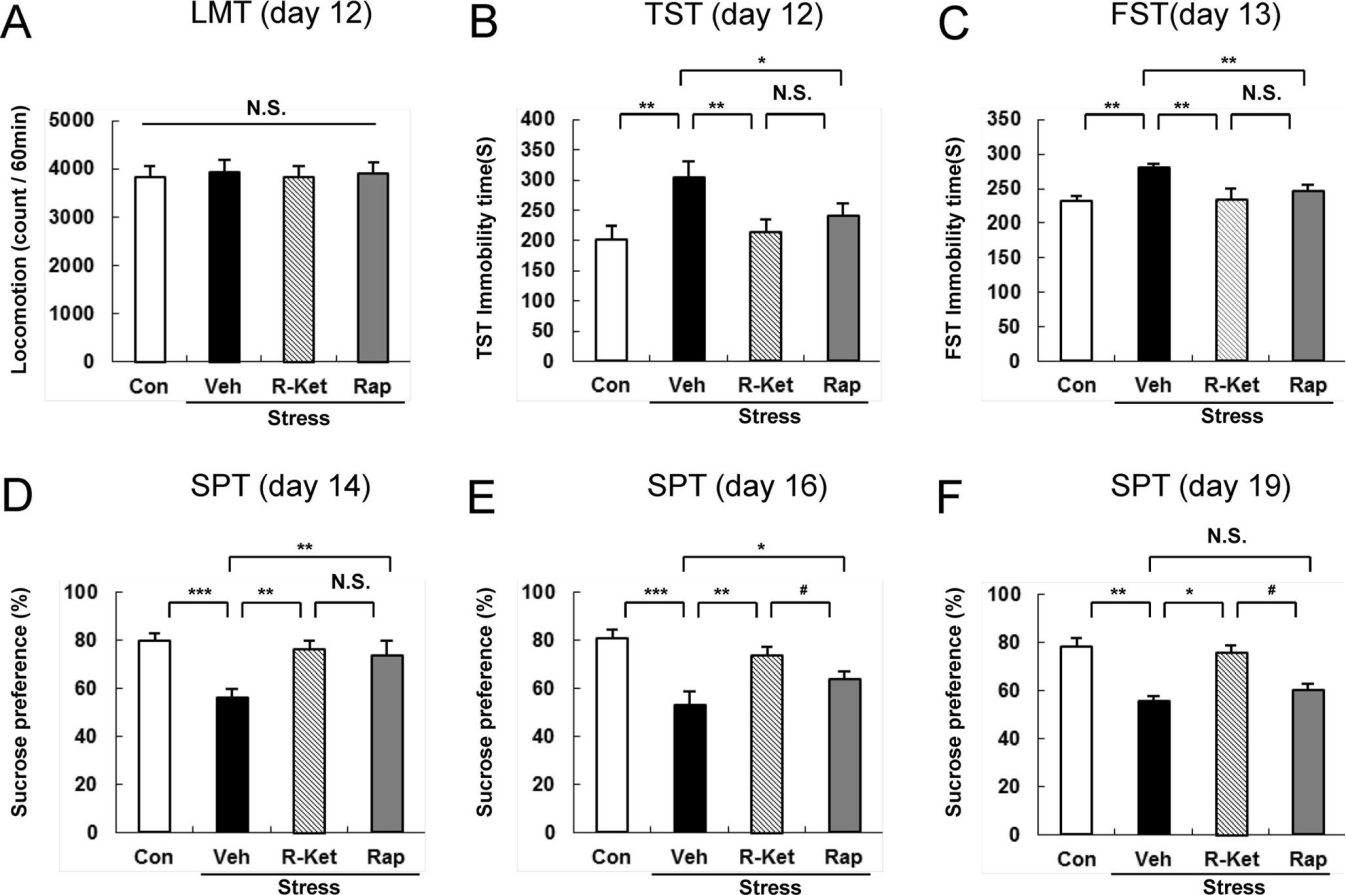
Effects of i.v. administration of *R*-ketamine and rapastinel in social defeat stress model. **a** LMT (day 12), **b** TST (day 12), **c** FST (day 13), **d** SPT (day 14), **e** SPT (day 16), and **f** SPT (day 19). The values represent the mean ± SEM (*n* = 9). *P < 0.05, **P < 0.01, ***P < 0.001 compared with the vehicle-treated stress group. ^#^ *P* < 0.05 compared with *R*-ketamine treated stress group. *N.S.* not significant, *Con* control, *Veh* vehicle, *R-Ket R*-ketamine, *Rap* rapastinel, *LMT* locomotion test, *TST* tail suspension test, *FST* forced swimming test, *SPT* 1 % sucrose preference test

In this study, we compared the effects of a single i.v. administration of *R*-ketamine and rapastinel in the social defeat stress model of depression (Fig. 1b). Locomotion showed no difference (*F*_3, 32_ = 0.014, *P* = 0.998) among the four groups (Fig. 6a). In the TST and FST, *R*-ketamine (3 mg/kg, i.v.) and rapastinel (3 mg/kg, i.v.) significantly attenuated the increased immobility times in susceptible mice (Fig. 6b, c). One-way ANOVA detected statistical significance in both the TST and FST (TST: *F*_3, 32_ = 4.541, *P* = 0.009; FST: *F*_3, 32_ = 4.656, *P* = 0.008) among the four groups (Fig. 6b, c). In the SPT, preference of mice after i.v. injection of *R*-ketamine was significantly higher (6D (day 14): *F*_3, 32_ = 9.422, *P* < 0.001, 6E (day 16): *F*_3, 32_ = 6.829, *P* = 0.001, 6F (day 19): *F*_3, 32_ = 4.118, *P* = 0.014) than that of the vehicle-treated group (Fig. 6d–f). In the SPT (days 16 and 19), efficacy of *R*-ketamine was significantly more potent than that of rapastinel (Fig. 6e, f). These behavioral data suggest that a single i.v. injection of *R*-ketamine and rapastinel promotes rapid antidepressant effects in the social defeat stress model and that *R*-ketamine produces longer lasting antidepressant effects compared to rapastinel.

## Discussion

The major findings of this study are that a single dose (i.p. and i.v.) of *R*-ketamine or rapastinel promoted a rapid antidepressant response in the social defeat stress model of depression and that *R*-ketamine produced longer lasting antidepressant effects than rapastinel. The rapid and sustained antidepressant effects of ketamine (or *R*-ketamine) in the social defeat stress model (Yang et al. 2015b; Zhang et al. 2015b; this study) are similar in time course to the therapeutic effects seen in patients with treatment-resistant depression and bipolar depression (Aan Het Rot et al. 2012; Zarate et al. 2006; Diazgranados et al. 2010; Zarate et al. 2012). To the best of our knowledge, this is the first report showing a comparison of antidepressant effects for *R*-ketamine and rapastinel in the social defeat stress model of depression.

We previously reported a marked reduction of BDNF protein in the PFC, DG, and CA3, but not CA1, of inflammation-induced depressed mice (Zhang et al. 2015a), social defeat stress model (Yang et al. 2015b; Zhang et al. 2015b), and learned helplessness rats (Shirayama et al. 2015; Yang et al. 2015a). In this study, we found a marked reduction of BDNF protein in the PFC, DG, and CA3, but not CA1, of susceptible mice after social defeat stress. In contrast, we found that inflammation and learned helplessness induced a marked increase in BDNF protein within the NAc (Zhang et al. 2015a; Yang et al. 2015a), consistent with higher BDNF levels in the NAc of susceptible mice following social defeat stress. The BDNF-TrkB pathway in the NAc plays a role in the depression phenotype (Nestler and Carlezon 2006; Ren et al, 2015; Yang et al. 2015a; Zhang et al. 2015a; 2015b). In this study, we also found that social defeat stress produced an opposing effect on BDNF protein levels in the PFC and hippocampus and NAc. Previously, it was reported that intra-VTA BDNF injections lead to depression-like behavior, while a blockade of BDNF activity in the NAc produced antidepressant-like effects (Nestler and Carlezon 2006). It is probable that social defeat stress causes decreased BDNF in the hippocampus and PFC, but increased BDNF in the NAc, resulting in depression-like behavior in mice.

We recently reported that TrkB agonist 7,8-DHF and TrkB antagonist ANA-12 showed antidepressant activity on inflammation (or social defeat stress)-induced depressive behavior, by normalizing altered dendritic spines in the PFC and hippocampus and NAc, respectively (Zhang et al. 2015a; 2015b). Furthermore, we also found that direct infusion of 7,8-DHF (but not ANA-12) into the hippocampus (CA3 and DG) and PFC and of ANA-12 (but not 7,8-DHF) into the NAc promoted antidepressant effects in the rat learned helplessness model (Shirayama et al. 2015), implying that stimulation at TrkB in the PFC, CA3, and DG, as well as blockade of TrkB in the NAc, conferred antidepressant effects. Therefore, it is likely that 7,8-DHF and ANA-12 act by normalizing altered BDNF-TrkB signaling in the PFC and hippocampus and NAc, respectively. In this study, we found that *R*-ketamine could attenuate reduced levels of BDNF protein in the PFC, CA3, and DG, but not NAc, 8 days after a single dose, consistent with previous reports (Yang et al. 2015b; Zhang et al. 2015b). Therefore, it is unlikely that BDNF-TrkB signaling in NAc is necessary to mediate the antidepressant effect of *R*-ketamine, although further studies are needed.

In this study, a single dose of *R*-ketamine, but not rapastinel, attenuated a marked increase levels of GluA1 (or PSD-95) proteins in the PFC, DG, and CA3, although both drugs showed a rapid antidepressant effect in the social defeat stress model. Considering the role of synaptogenesis in the sustained antidepressant effect of ketamine (Duman and Aghajanian 2012; Ohgi et al. 2015), it seems that long-lasting increases of GluA1 and PSD-95 in the PFC, DG, and CA3 may underlie *R*-ketamine’s long-lasting action. Nonetheless, further detailed studies on the role of synaptogenesis in this sustained antidepressant response are needed.

Burgdorf et al. (2013) reported that antidepressant effects of rapastinel (3 mg/kg, i.v.) were similar to ketamine (10 mg/kg, i.v.)-induced antidepressant effects in multiple rat models of depression. In this study, we used the lower dose (3 mg/kg) of *R*-ketamine in the social defeat stress model. It is noteworthy that *R*-ketamine (3 mg/kg, i.v.) has longer lasting antidepressant effect than rapastinel (3 mg/kg, i.v.). In addition, we also found that antidepressant effects of the NMDA receptor antagonist MK-801 could not detected 7 days after a single dose although MK-801 showed rapid antidepressant effect in the social defeat stress model (Yang et al. submitted). Taken together, it is unlikely that the NMDA receptor inhibition may play a key role in the sustained antidepressant effects of ketamine.

The pharmacokinetic profile of ketamine in male C57/B6 mice has been reported. The half-life of ketamine in mouse plasma is approximately 30 min (Sato et al. 2004), suggesting a possible rapid clearance of *R*-ketamine from the body. To date, the pharmacokinetic profile of rapastinel in mice is unknown. In this study, we detected a sustained antidepressant response 7 days after a single dose of *R*-ketamine or rapastinel although *R*-ketamine’s sustained antidepressant effect was longer than that of rapastinel. Taken together, it is unlikely that this differential antidepressant effect between *R*-ketamine and rapastinel is due to differences in pharmacokinetic profiles.

In conclusion, this study shows that a single dose of *R*-ketamine or rapastinel can produce rapid antidepressant effects in the social defeat stress model of depression and that *R*-ketamine elicits a longer lasting antidepressant effect than rapastinel. Furthermore, it is likely that increased synaptogenesis in the PFC, DG, and CA3 of the hippocampus may be involved in the sustained antidepressant response of *R*-ketamine.
